# Synthesis of photo-reactive poly (vinyl alcohol) and construction of scaffold-free cartilage like pellets *in vitro*

**DOI:** 10.1093/rb/rby009

**Published:** 2018-05-03

**Authors:** Bao Li, Yongli Gao, Likun Guo, Yujiang Fan, Naoki Kawazoe, Hongsong Fan, Xingdong Zhang, Guoping Chen

**Affiliations:** 1 National Engineering Research Center for Biomaterials, Sichuan University, 29 Wangjiang Road, Chengdu, Sichuan 610064, China; 2 Research Center for Functional Materials, National Institute for Materials Science, 1-1 Namiki, Tsukuba, Ibaraki 305-0044, Japan

**Keywords:** surface modification, photo-reactive poly (vinyl alcohol), chondrogenic differentiation

## Abstract

Photo-reactive poly(vinyl alcohol) (PRPVA) was synthesized by introduction of phenyl azido groups into poly(vinyl alcohol) (PVA) and applied for surface modification. PRPVA was grafted onto cell culture plate surface homogeneously or in a micropattern. Human mesenchymal stem cells (hMSCs) cultured on cell culture plate surface and PVA-modified surface showed different behaviors. Cells adhered and spread well on cell culture plate surface, while they did not adhere on PVA-grafted surface at all. When hMSCs were cultured on PVA-micropatterned surface, they formed a cell micropattern. Cells formed pellets after cultured on PVA homogeneously modified surface in chondrogenic induction medium for 2 weeks. The pellets were positively stained by hematoxylin/eosin, safranin-O/fast green and toluidin blue, and they were also stained brown by Type II collagen and proteoglycan immunohistological staining. Real-time PCR analysis was conducted to investigate the expression of colI, colII, colX, aggrecan and sox9 mRNA. Results of gene expression were in agreement with those of histological and immunohistological observations. These results indicated that hMSCs cultured on PVA-modified surface performed chondrogenic differentiation, and it was possible to construct scaffold-free cartilage like pellets with PVA-modified surface *in vitro*.

## Introduction

Mesenchymal stem cells (MSCs) are a unique class of multipotent cells; they can be easily extracted from bone marrow, adipose tissue, umbilical cord tissue and so on. When cultured in proper environment, MSCs can differentiate into chondrocytes, osteoblasts, adipocytes, skeletal muscle cells and cardiac muscle cells etc. [1, 2]. MSCs have been widely applied in tissue engineering due to these advantages, especially in cartilage tissue engineering [3–6].

Chondrogenic differentiation of MSCs is affected by many factors, such as bioactive factors, cells density, materials surface properties etc. [6, 7]. Growth factors, e.g. insulin like growth factor-1, fibroblast growth factors, transforming growth factor-β (TGF-β) and bone morphogenetic proteins have been proved to participate in chondrogenic induction, synthesis of extracellular matrix, and maintenance of chondrocytes phenotype during chondrogenic differentiation of MSCs [3, 8]. Most of studies indicate that high cell density improves chondrogenic differentiation of MSCs [6, 7], so that the micromass or pellet culture with chondrogenic induction supplement is often chosen to confirm the potential of stem cells to differentiate into chondrocytes. Surface properties of scaffold, such as surface chemistry, nano or micro-structure, roughness and electrostatic property, have important effect on cell behaviors, which directly affect cell attachment, proliferation, migration, extracellular matrix secretion and differentiation [9–16].

In our previous study, polyallylamine (PAAm), poly(ethylene glycol) (PEG) and poly(acrylic acid) were selected to modify cell culture plate surface and their effects on the chondrogenic differentiation of hMSCs were investigated. Results indicated that the chondrogenic differentiation of hMSCs was promoted when cells were cultured on PEG and PAAm modified surfaces [9]. However, it was difficult to immobilize neutral photo-reactive PEG on cell culture plate surface homogeneously because of the difficulty in preparing a homogeneous film with PEG due to its low-molecular weight and lack of functional groups in PEG molecules.

PVA, another neutral polymer, is a kind of water-soluble polyhydroxyl polymer with low toxicity, good biocompatibility and excellent film-forming ability, it has been widely applied in the field of tissue engineering [17, 18], drug delivery [19, 20], food packaging [21] etc. There are abundant hydroxyl groups on the side chain of PVA which can be easily modified to improve its chemical and biological properties for application in tissue engineering [22–26].

Taken what has been discussed above into consideration, and combining excellent film-forming ability of PVA with the property to support chondrogenic differentiation of PEG-modified surface, in this study, another kind of neutral photo-reactive polymer, photo-reactive PVA (PRPVA), was designed and fabricated to modify cell culture plate surface; then its effect on the adhesion and chondrogenic differentiation of hMSCs was investigated; finally, scaffold-free cartilage like pellets was constructed on PVA-modified surface.

## Materials and methods

### Materials

4-(1-pyrrolidinyl) pyridine and PVA (degree of polymerization was 1000, completely hydrolyzed) were purchased from Wako Pure Chemical Industries Ltd. 4-azidobenzoic acid was obtained from Tokyo Kasei Kogyo Co. Ltd. Dicyclohexylcarbodiimede (DCC) was a product of Peptide Institute. Inc. All of these materials and reagents were used as received without further purification.

### Preparation and purification of PRPVA

PRPVA was prepared by the reaction of PVA and 4-azidobenzoic acid [27]. A total of 2 ml dimethyl sulphoxide (DMSO) solution dissolving 243.3 mg DCC was added to DMSO solution dissolving 193.9 mg 4-azidobenzoic acid under stirring in the dark, then 2 ml DMSO solution containing 19.6 mg 4-(1-Pyrrolidinyl) pyridine was dropped to the above mixture under stirring. After 10 min, 8 ml DMSO solution dissolving 100 mg of PVA was added, and the reaction proceeded overnight. During the reaction of a hydroxyl group and an acid group, DCC was converted to dicyclohexylurea (DCU) and precipitated out of the solution. After filtration of DCU, the filtrate was further purified by dialysis. At last, the sample was freeze-dried and preserved at 4°C in the dark until use.

### Characterization of PRPVA

^1^H-NMR spectrum was recorded on a JEOL EX-300 NMR spectrometer running at 300 Hz for ^1^H-NMR measurement. D_2_O was used as solvent for both PVA and PRPVA, and tetramethylsilane was used as an internal standard. The content of azidophenyl groups in PRPVA was calculated from the peak intensity of the azidophenyl protons and those of the methylidyne protons in ^1^H-NMR spectrum.

### Preparation and characterization of PVA micropattern

To confirm whether the PRPVA can be grafted onto cell culture plate surface, the PVA micropattern was prepared. PRPVA was dissolved in MilliQ water, and the aqueous solution was cast on polystyrene discs and dried in air. The PRPVA-coated discs were covered by a photomask and UV irradiated (1 × 10^5^uJ/cm^2^, 60 s) at a distance of 15 cm. After UV irradiation, the PVA-modified discs were washed with MilliQ water. The obtained PVA pattern was observed with optical microscopy to investigate the integrity of PVA micropattern, and the height of grafted PVA was measured by scanning probe microscopy (SPM) [27].

### Grafting of PRPVA on the surface of cell culture plate

The PRPVA was dissolved in MilliQ water and the solution was cast in every well of a six-well cell culture plate and dried at room temperature in the dark. The plate was irradiated at an irradiation intensity of 10^5^ uJ/cm^2^ from the distance of 15 cm for 1 min to graft PRPVA on the surface of six-well cell culture plate. After irradiation, the plate was washed by sonicating to get rid of unreacted polymer. After washing, the PVA homogeneously grafted plate was sterilized and stored at 4°C until use.

### Cells culture

hMSCs were available from Osiris (Worthington Biochemical, Lakewood, NJ) at Passage 2. Cells were cultured using the proliferation medium from Osiris. hMSCs were used at Passage 4. The cells were collected and washed with DMEM serum-free medium, and then suspended in the medium with a cell density of 7.0 × 10^5^ cells/ml. Cells suspension was placed to PVA-modified and non-modified cell culture plate (1 ml/well). 5 ml DMEM serum-free medium (as control) or chondrogenic differentiation medium was added to each well, and the cells were cultured for 2 weeks. The chondrogenic differentiation medium was composed of serum-free DMEM solution containing 584 mg/l glutamine, 4500 mg/l glucose, 100 μg/ml streptomycin, 100 U/ml penicillin, 0.1 mM nonessential amino acids, 50 mg/l ascorbic acid, 0.4 mM proline, 10^−7^ M dexamethasone and 10 ng/ml TGF-β3. The cell culture medium was refreshed every 3 days. The medium was changed carefully to ensure that the cell pellets formed during culture were not replaced. Cell pellets were harvested for histological examination and gene expression analysis after cultured for 2 weeks.

To observe the effect of PVA on hMSCs adhesion directly, hMSCs were cultured on PVA-micropatterned surface in hMSCs proliferation medium. At proper timepoint, the morphology of cells was observed by optical microscopy.

### Histology and immunohistochemistry

The pellets formed on cell culture plate surface and PVA-modified surface were fixed, embedded and sectioned. The sections were stained by toluidin blue and safranin-O/fast green staining to detect cartilaginous specific matrix secretion and stained by hematoxylin and eosin (H&E) staining to observe cell morphology.

Cartilaginous proteoglycan, Type II collagen and Type I collagen were immunohistologically stained. Mouse anti-human Type II collagen monoclonal antibody, rabbit anti-human Type I collagen antibody and mouse anti-human cartilage proteoglycan monoclonal antibody were used, and the process for staining was the same as previously described in [28].

### Biochemical analysis

Cell pellets formed when hMSCs were cultured on cell culture plate surface and PVA-modified surface in the chondrogenic induction medium for 2 weeks. The DNA content in the pellets was determined by the DNA analysis kit (Sigma-Aldrich, St Louis, MO, USA) [29], and sulfated glycosaminoglycans (GAGs) content was detected according to a sulfated GAG assay kit, BlyscanTM (Biocolor Ltd., Newtownabbey, Northern Ireland) [30].

### RNA isolation and real-time PCR analysis

Cell pellets formed on cell culture plate surface and PVA-modified surface in the control and chongdrogenic differentiation medium for 2 weeks were washed with PBS, frozen in liquid nitrogen and crushed into powder. The powder was collected and dissolved in 1 ml of Isogen reagent, and the RNA was isolated. DNase-treated RNA was treated with RQ1 RNase-free DNase before it was converted to cDNA. Real-time PCR was amplified for Type I collagen, Type II collagen, Type X collagen, aggrecan, sox9 and GAPDH. The reaction was conducted with 300 nM PCR primer, 150 nM PCR probe, 1 μl cDNA and TaqMan Universal PCR Master Mix. The reaction was run with a Bio-Rad iCycler for 40 cycles. The expression level of each gene was normalized to GAPDH. The data were analyzed by Bio-Rad iCycker software. The sequences for primer and probe were designed according to Martin *et al*. [31] and Schaefer *et al.* [32].

## Results and discussion

### Preparation and characterization of PRPVA

**Figure 1:**
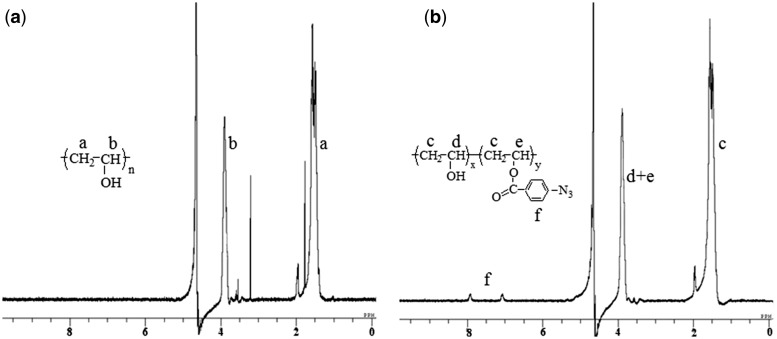
^1^H-NMR spectra of PVA **(a)** and PRPVA **(b)** in D_2_O

PVA is a kind of water-soluble polyhydroxyl polymer. It has a good film-forming ability and it strongly resists to the adhesion of proteins and cells. PVA has found some applications in the field of protein and cell adhesion, cell–matrix and cell–cell interactions and cartilage tissue engineering [17, 18, 33, 34]. In this study, photo-reactive groups were introduced into the side chain of PVA by the reaction of PVA and 4-azidobenzoic acid at the presence of DCC and 4-(1-Pyrrolidinyl) pyridine in DMSO solution at room temperature. The byproduct DCU was precipitated and filtered, and the final product was purified by dialysis against MilliQ water. The introduction of azidophenyl group into the side chain of PVA was confirmed by ^1^H-NMR spectrum, as shown in Fig. 1, the azidophenyl proton appeared in the ^1^H-NMR spectrum at 7.07 and 7.93 ppm, methine and methene protons appeared at 3.9 ppm and 1.5 ppm, respectively. The content of azidophenyl group in the photo-reactive polymer was about 2% as calculated from the intensity of the phenylazido protons and the intensity of methine protons in ^1^H-NMR spectrum.

### Surface grafting and micropattern preparation

**Figure 2:**
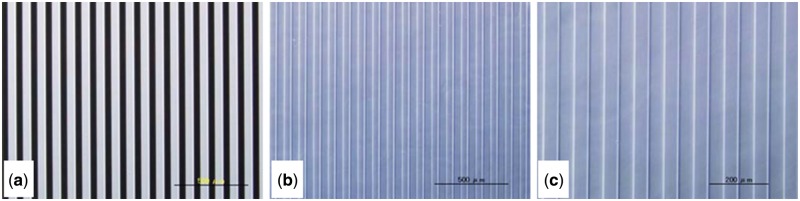
Optical microscope images of photomask **(a)**, PVA micropattern with low **(b)** and high **(c)** magnification. Scale bar is 500 μm for (a) and (b); 200 μm for (c)

To make sure whether or not the PRPVA could be grafted onto the surface of cell culture plate, a photomask was used to prepare PVA micropattern. At first, PRPVA was cast on cell culture plate surface, and dried in air at room temperature. UV-irradiation was performed after a photomask was placed on the surface of PRPVA-coated cell culture plate. The PRPVA in the UV-irradiated area generated biradicals and was immobilized on cell culture plate surface, but they would not be grafted in other areas and be washed off. Figure 2 shows the photomask (Fig. 2a) and the PRPVA micropattern (Fig. 2b and c). It indicated that the PRPVA was grafted onto the cell culture plate surface in the same pattern with the photomask, and the micropattern with a good integrity. The height of grafted PVA was 93.16 ± 8.66 nm in air and 137.97 ± 22.01 nm in MilliQ water as measured by SPM.

### Cells morphology on PVA homogeneously grafted and micropatterned surfaces

**Figure 3:**
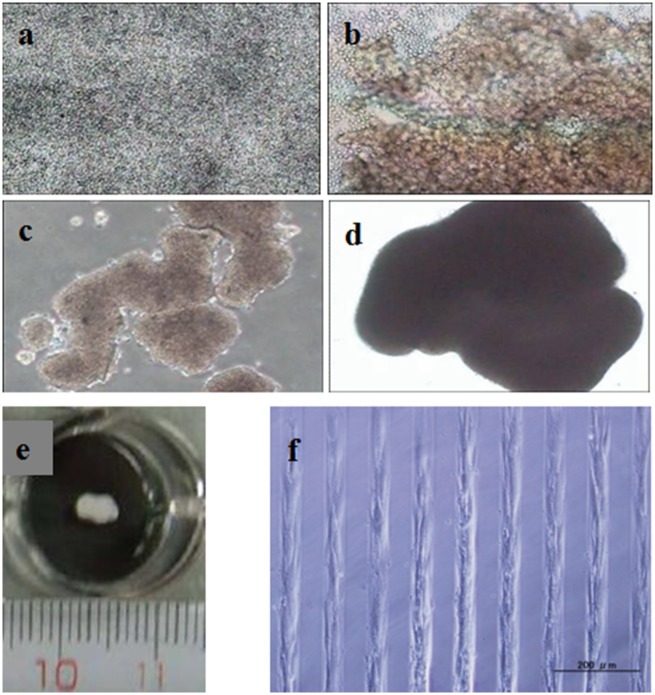
Cells morphology cultured on PVA-modified surface in chondrogenic induction medium after 0.5 h **(a)**, 3 h **(b)**, 1 day **(c)**, 2 days **(d)**, 14 days **(e)** and cells morphology cultured on PVA micropatterned surface for 1 day **(f)**

hMSCs cultured on PVA modified and cell culture plate surfaces in chondrogenic differentiation medium showed different morphology. The cells attached and spread on cell culture plate surface when cultured in differentiation medium for 3 h, and became confluent after being cultured for 3 days. Cells migrated after cultured for several days, and there were some areas not covered by cells, with time going on the cells would aggregate into pellets. Although cells did not attach on the PVA-modified surface at all (Fig. 3a), they showed a cloudy-like morphology after seeded in differentiation medium for 3 h (Fig. 3b). Cells would aggregate into pellets with the prolongation of culture time, as shown in Fig. 3c–e. Cells morphology cultured on PVA-modified surface was similar to that cultured on PEG-modified surface [9]. When hMSCs were cultured on PVA micropatterned surface, cells adhered only on cell culture plate—polystyrene areas, and they formed a micropattern (Fig. 3f). It further confirmed the resistance of PVA to cells and proteins adhesion. Based on this property of PVA, we assumed that PVA-modified surface could provide a 3D microenvironment for hMSCs to freely communicate with each other and fully interact with nutrition and growth factors whether addition or secreted by cells themselves.

### Histological staining, immunohistological staining and biochemical analysis

Secretion of cartilaginous-specific ECM was an important characterization for chondrogenic differentiation of MSCs. In this study, the pellets were detected qualitatively by histological and immunohistological staining and quantitatively by GAGs and DNA content analysis.

**Figure 4:**
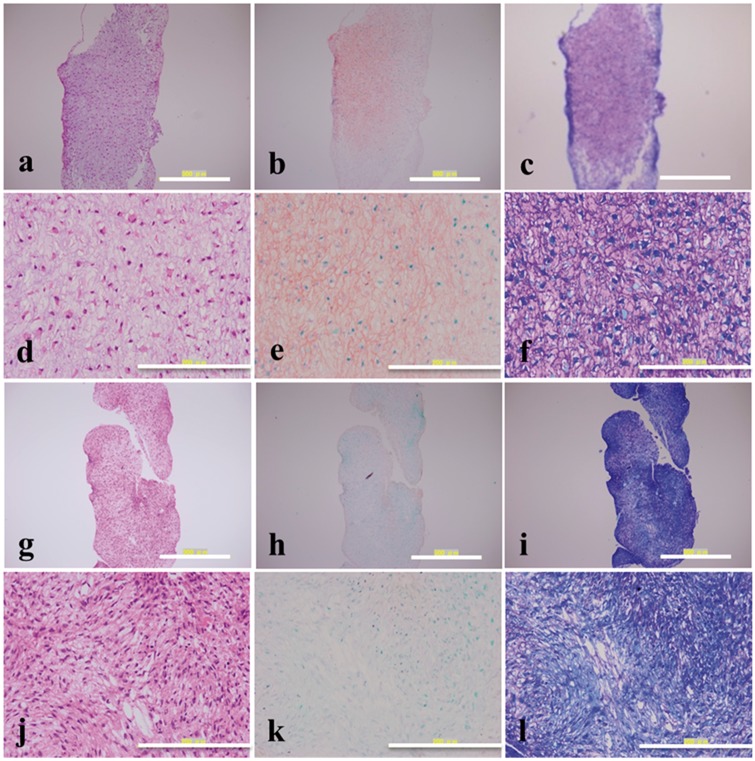
H&E (**a**, **d**, **g**, **j**), safranin-O/fast green (**b**, **e**, **h**, **k**) and toluidin blue (**c**, **f**, **i**, **l**) staining of pellet formed on PVA-modified surface **(a–f)** and cell culture plate surface **(g–l)** cultured in chondrogenic induction medium for 2 weeks. Scale bar is 500 μm for (a–c) and (g–i); 200 μm for (d–f) and (j–l)

The cells cultured on both cell culture plate surface and PVA-modified surface formed pellets when cultured in differentiation medium for 2 weeks. The obtained pellets were stained by H&E, safranin-O/fast green and toluidin blue staining to observe cell morphology and cartilaginous specific extracellular matrix synthesis. In H&E staining, cells in pellets formed on PVA-modified surface in chondrogenic differentiation medium showed a round morphology (Fig. 4a and d), while cells in pellets formed on cell culture plate surface in the same condition showed a spindle, fibroblast like morphology (Fig. 4g and j). Cells in pellets formed on PVA-modified surface in chondrogenic differentiation medium were stained red with safranin-O/fast green staining (Fig. 4b and e) and purple with toluidin blue staining (Fig. 4c and f), which indicated that there were abundant GAGs distributed around cells. However, cells in pellets formed on cell culture plate surface in the same culture condition did not show positive staining for them (Fig. 4h, k, i and l). These results demonstrated that PVA-modified surface was beneficial for hMSCs to perform chondrogenic differentiation, and the extent of chondrogenic differentiation for hMSCs was higher compared with that on cell culture plate surface.

**Figure 5:**
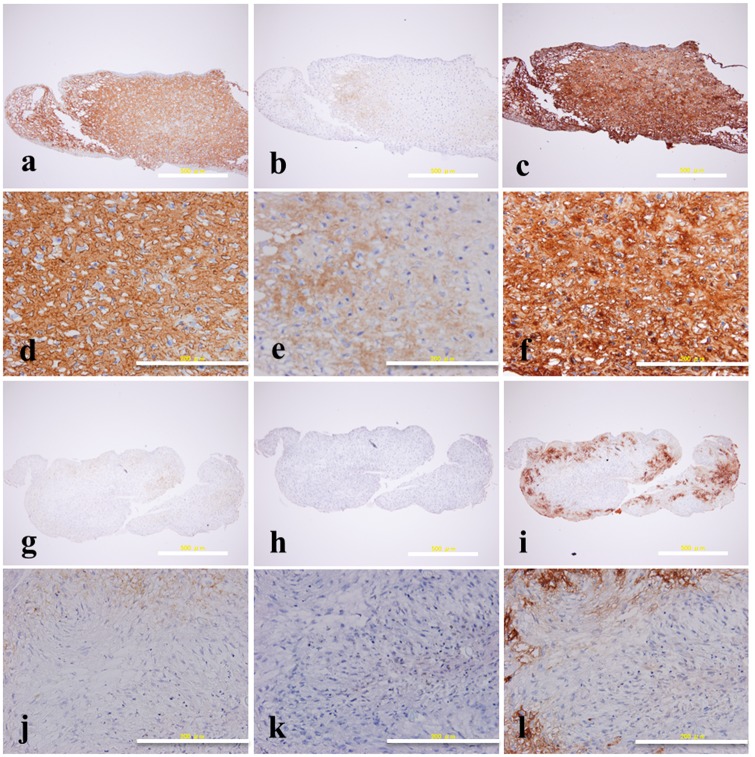
Immunostaining of Type I collagen (**a**, **d**, **g**, **j**), Type II collagen (**b**, **e**, **h**, **k**) and cartilage proteoglycan (**c**, **f**, **i**, **l**) of pellet formed on PVA-modified surface **(a–f)** and cell culture plate surface **(g–l)** cultured in chondrogenic induction medium for 2 weeks. Scale bar is 500 μm for (a–c) and (g–i); 200 μm for (d–f) and (j–l)

Matrix protein analysis for cartilaginous proteoglycan, Type II collagen and Type I collagen was performed by immunohistological staining, and the results were showed in Fig. 5. Cells in pellets formed on PVA-modified surface stained brown with Type I collagen (Fig. 5a and d), Type II collagen (Fig. 5b and e) and cartilaginous proteoglycan (Fig. 5c and f) immunohistological staining, while cells in pellets formed on cell culture plate surface did not show obvious positive staining for them (Fig. 5g–l). These results were in accordance with those of toluidin blue, safranin-O/fast green and H&E staining, and indicated that there was abundant cartilaginous ECM secretion when hMSCs were cultured on PVA-modified surface.

The contents of DNA and sulfated GAGs in pellets were analyzed to calculate the secretion of cartilaginous ECM quantitatively, and results were shown in Table 1. DNA contents in pellets formed on PVA-modified, and cell culture plate surfaces were 9.28 ± 0.37, 12.42 ± 0.41 μg(DNA)/mg(pellets), respectively. GAGs contents in pellets formed on PVA-modified, and cell culture plate surfaces were 34.76 ± 1.02, 5.60 ± 0.76 μg(GAGs)/mg(pellets), respectively. The ratio of GAGs to DNA in pellets formed on PVA-modified, and cell culture plate surfaces were 3.75 ± 0.11, 0.45 ± 0.06, respectively. Pellets formed on the PVA-modified surface produced significantly more GAGs than did those on cell culture plate surface.

**Table 1:** DNA and GAGs content in cell pellets cultured on PVA-modified and cells culture plate surfaces for 2 weeks

	PVA-modified surfaces	Cell culture plate surfaces
DNA (μg DNA/mg pellets)	9.28 ± 0.37	12.42 ± 0.41
GAGs (μg DNA/mg pellets)	34.76 ± 1.02	5.60 ± 0.76
Ratio of GAGs/DNA	3.75 ± 0.11	0.45 ± 0.06

### Gene expression

**Figure 6:**
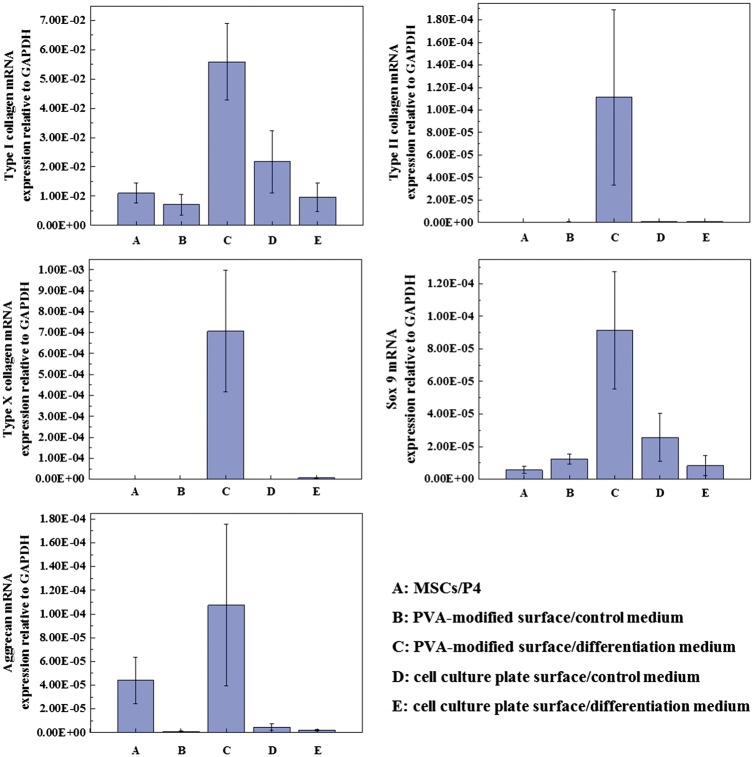
Real-time PCR results of mRNA expression of Type I collagen, Type II collagen, Type X collagen, sox9 and aggrecan of hMSCs cultured on PVA-modified surface and cell culture plate surface in the control or differentiation medium for 2 weeks. The data are normalized to GAPDH value

Real-time PCR analysis was conducted to investigate the expression of Type I collagen, Type II collagen, Type X collagen, aggrecan and sox9 mRNA by the cells cultured on PVA-modified surface in control and chondrogenic differentiation medium for 2 weeks, and the results were shown in Fig. 6. hMSCs of Passage 4 expressed genes encoding Type I collagen, a low level of sox9 and aggrecan, while they did not express genes encoding Type X collagen and Type II collagen. The hMSCs cultured on PVA-modified surface in chondrogenic differentiation medium expressed all of these genes; especially genes encoding aggrecan, sox9, Type II collagen and Type X collagen were upregulated. When hMSCs cultured on PVA-modified surface in control medium and on cell culture plate surface in both control and differentiation medium for 2 weeks, genes encoding Type I collagen and only a low level of sox9 were expressed, while the expression of genes encoding Type X collagen, Type II collagen and aggrecan was almost undetectable. Cartilaginous specific genes of aggrecan and Type II collagen were expressed when hMSCs were cultured on PVA-modified surface in chondrogenic differentiation medium. These results were in agreement with histological and immunohistological observations, and demonstrated that PVA-modified surface provided a proper microenvironment for hMSCs to secrete cartilaginous ECMs.

Suitable microenvironment, high cell density, freely cell–cell contact and communication are benefit for the construction of cartilage tissue. Pellet culture of MSCs was usually applied to detect the chondrogenisis ability of MSCs. In this study, hMSCs were cultured on PVA-modified surface and suspended freely in the medium. Cells communicated with each other and interacted with nutrient and growth factors freely, and they got together and formed cloudy-like morphology by themselves. With prolongation of culture duration, the cloudy-like cells aggregated into incompact pellets. The round morphology of cells and the secretion of cartilaginous ECM such as sulfated GAGs and Type II collagen are main characterization for MSCs to perform chondrogenic differentiation [35]. Almost all of cells in the pellets formed on PVA-modified surfaces showed round morphology, and rounded by cartilaginous extracellular matrix as characterized by histological and immunohistological staining. The size of pellets was related with cells number, and two or more pellets could grow into a big one when they contacted with each other. The incomplete differentiation of hMSCs and the inadequate contact between cells and nutrients could be avoided by culturing hMSCs on PVA-modified surface. This study provided a simple and meaningful method for fabrication of cartilage-like pellet *in vitro*. Scaffold materials were not applied during the process of cartilage-like pellets formation, which avoided issues brought by scaffolds, e.g. cells inhomogeneous distribution in scaffold, and the effect of degradation products of biodegradable materials on cell activity and behaviors etc. [4].

## Conclusions

A kind of neutral photo-reactive polymer, PRPVA, was prepared by a one-step reaction method, and it could be grafted onto cell culture plate surface homogeneously or in a micropattern. The PVA-modified surface provided a suitable microenvironment for chondrogenic differentiation of hMSCs, and scaffold-free cartilage like pellets were constructed *in vitro*. This study provided a convenient method for scaffold-free cartilage like pellets construction, and implied that the PRPVA had a potential application in cartilage tissue engineering.
